# Combined and progressive effect of frailty and sarcopenia on the risk of cardiovascular disease in middle-aged and older adults

**DOI:** 10.7189/jogh.15.04299

**Published:** 2025-10-31

**Authors:** Menglu Liu, Jiawen Li, Kailun Yan, Kexin Zhang, Man Wang, Tianyu Li, Jidong Guo, Nuerbahati Heisha, Yuejin Yang, Jinqing Yuan, Yunqing Ye, Xueyan Zhao

**Affiliations:** 1 National Clinical Research Center for Cardiovascular Diseases, State Key Laboratory of Cardiovascular Disease, Fu Wai Hospital, National Center for Cardiovascular Diseases, Chinese Academy of Medical Sciences and Peking Union Medical College, Ward 16, Beijing, China; 2 Shihezi People's Hospital, Department of Cardiology, Shihezi, China

## Abstract

**Background:**

Frailty and sarcopenia are two related conditions, but how they lead to cardiovascular disease (CVD) remain unclear. The study aimed to investigate the combined and progressive effect of them.

**Methods:**

The data were from China Health and Retirement Longitudinal Study (CHARLS). Frailty status was evaluated by the Rockwood frailty index and sarcopenia status was classified according to the Asia Working Group for Sarcopenia 2019.

**Results:**

A total of 7187 participants aged over 45 years (49.41% male; mean age 57.38 ± 8.41 years) were enrolled in 2011. Among them, 4265 individuals without CVD events between 2011 and 2015 were included in the analysis of dynamic changes. Over the nine-year period, both frailty and sarcopenia status were independently associated with an increased risk of CVD. A significant interaction for CVD was observed between frailty and sarcopenia status (*P* for interaction <0.001). Furthermore, the coexistence of frailty and sarcopenia was associated with the highest risk of incident CVD (hazard ratios (HR) = 2.18; 95% confidence interval (CI) = 1.81–2.62), indicating that individuals with both conditions had more than double the risk of developing CVD, highlighting a substantially elevated cardiovascular vulnerability. Additionally, frailty was found to statistically mediate approximately 35.38% of the association between sarcopenia and CVD. Regarding dynamic changes, individuals who remained frail or experienced worsening sarcopenia were associated with the highest incidence of CVD, with HRs of 3.29 (95% CI = 2.44–4.45) and 1.56 (95% CI = 1.28–1.90), respectively, compared to those who remained robust or non-sarcopenic. These findings indicate that persistent frailty and deteriorating muscle health substantially increase CVD risk.

**Conclusions:**

The study indicated there was a combined and progressive effect of frailty and sarcopenia on CVD. These findings highlighted the importance of identifying and improving sarcopenia and frailty.

With global population ageing intensifying, frailty and sarcopenia, as the common geriatric syndromes, impose a significant public health burden on ageing societies [1–3]. Frailty reflects a decline in physiological reserve and resilience, while sarcopenia involves the progressive loss of skeletal muscle mass and function [4–7].

Cardiovascular disease (CVD) remains a leading cause of morbidity and mortality globally, and its burden increases markedly with age [8,9]. In recent years, growing attention has been directed toward two age-related conditions – frailty and sarcopenia – that may contribute to the onset and progression of CVD. Longitudinal cohort studies from Europe, the USA, and China consistently demonstrate that frailty is associated with an elevated risk of incident CVD [10,11]. Similarly, data from a large Chinese cohort indicated that both possible sarcopenia and sarcopenia were linked to elevated CVD risks [12]. However, these associations are largely derived from observational studies, which may be influenced by residual confounding, measurement error, and reverse causation, limiting causal inference.

To address these limitations, Mendelian randomisation studies have provided more robust evidence supporting potential causal relationships. Genetically predicted reductions in muscle mass and muscle strength – key features of sarcopenia – have been associated with increased risks of coronary artery disease and heart failure [13]. Likewise, higher genetically determined frailty index (FI) has been linked to greater susceptibility to myocardial infarction and cardiovascular mortality, suggesting frailty may also play a causal role in CVD development [14]. Although Mendelian randomisation provides stronger causal inference than traditional observational studies, the biological mechanisms through which frailty and sarcopenia impact CVD remain incompletely understood.

Although frailty and sarcopenia are two common clinical syndromes in older adults, they share many similarities in terms of aetiology and symptoms [15]. There is significant overlap between the two in terms of mechanisms, clinical outcomes, and interventions. Multiple international studies have shown that most patients with sarcopenia exhibit frailty. Similarly, frail older adults are at higher risk of developing sarcopenia [16]. Chronic inflammation can lead to sarcopenia by reducing IGF-1, damaging mitochondria, and lowering amino acid uptake. As muscle function deteriorates, it accelerates frailty, which further impairs physical function, metabolism, and vascular health, ultimately increasing the risk of CVD [17]. Therefore, these two conditions often coexist in individuals and influence each other [18]. However, existing studies have mostly focused on the individual effects of frailty or sarcopenia, overlooking the combined impact they have on CVD.

Research has shown that frailty and sarcopenia can both be reversed with timely intervention and guidance [19,20]. This reversibility highlights the importance of tracking their dynamic changes rather than relying solely on static assessments. Monitoring transitions in frailty and sarcopenia over time can enhance our understanding of ageing trajectories, enable earlier identification of individuals at elevated cardiovascular risk, and guide timely preventive measures before irreversible damage occurs [21].

Therefore, the present study utilised data from the China Health and Retirement Longitudinal Study (CHARLS) to examine the combined and progressive impact of frailty and sarcopenia on CVD in middle-aged and older adults. The findings aim to support early screening and intervention strategies to reduce the burden of CVD in ageing populations.

## METHODS

### Study population

The CHARLS study (available at http://charls.pku.edu.cn/) is a national cohort study focusing on adults aged 45 and older in China. Participants were recruited from rural and urban areas across 150 counties or districts in 28 provinces of China. The study was initiated in 2011 (Wave 1) and has since conducted follow-up assessments every two years, including Waves 2 through 5 in 2013, 2015, 2018, and 2020, respectively. Detailed information about CHARLS had been published in previous research [22,23]. The CHARLS research was approved by Peking University's IRB. Participants provided written informed consent before engaging in the study. The investigation adhered to the STROBE guidelines for reporting observational studies in epidemiology (Checklist S1 in the **Online Supplementary Document**). This study included 7187 individuals in a longitudinal analysis, and the selection process is illustrated (Figure S1 in the **Online Supplementary Document**).

### Assessment of frailty

Frailty was evaluated using the Rockwood FI, which in our study was constructed from 28 baseline items reflecting the accumulation of various age-related health deficits (Table S1 in the **Online Supplementary Document**). Following prior research conventions, frailty status was grouped into three categories: robust (FI ≤ 0.10), pre-frail (0.10 < FI < 0.25), and frail (FI ≥ 0.25) [24–26].

### Assessment of sarcopenia

Sarcopenia status was evaluated using the AWGS 2019 algorithm, which incorporates three key components: muscle strength, appendicular skeletal muscle mass (ASM), and physical performance [27].

Handgrip strength was measured using a Yuejian WL-1000 dynamometer, with participants instructed to squeeze the dynamometer with maximum effort using both their dominant and non-dominant hands. A threshold of <18 kg for women and <28 kg for men was used to define low muscle strength [27].

This research used an anthropometric equation to estimate Appendicular Skeletal Muscle (ASM) mass, which is a practical and cost-effective alternative to more precise but expensive methods like dual-energy x-ray absorptiometry (DXA). This equation has been validated and widely used, particularly in the Chinese population, where it shows strong agreement with DXA measurements [6].

ASM = 0.193 × weight(kg) + 0.107*height(cm) – 4.157 × gender-0.037 × age(years) –2.631

Weight and height were measured using an Omron HN-286 scale and a Seca 213 stadiometer, respectively. Gender was coded as 1 for males and 2 for females. Height-adjusted muscle mass (ASM/Ht^2^) was derived by dividing ASM by height squared. The cutoff values for low muscle mass were based on sex-specific lower 20th percentiles of ASM/Ht^2^ in the study population: 5.4 kg/m^2^ for females and 7.0 kg/m^2^ for males [28,29].

The study evaluated physical performance using three tests: gait speed, the chair stand test, and the short physical performance battery (SPPB) test. The cutoff for low physical performance is defined as a gait speed <1.0 m/s, five chair stand tests ≥12 seconds, or an SPPB score ≤9 [27]. More details had been described in previous research [30].

Possible sarcopenia is defined as low muscle strength or low physical performance. Sarcopenia based on the presence of low muscle mass along with either low muscle strength or low physical performance. In our analysis, possible sarcopenia and sarcopenia participants were merged into one category: sarcopenia for analysis.

### Covariates

The covariates included age, sex, marital status, living place, education, hukou, smoking status, drinking status, body mass index (BMI), haemoglobin (HB), hypertension, diabetes mellitus, dyslipidaemia, and kidney diseases. Marital status was categorised into two groups: married and others (separated, divorced, unmarried, or widowed). Education was grouped into two levels: primary school or below and high school or above. Hukou was categorised into two groups: agriculture and others (non-agriculture, unified residence and do not have hukou). Smoking status was categorised as never, former, or current. Drinking status was classified as never or ever. Hypertension is characterised by a systolic blood pressure of ≥140 mm Hg, a diastolic blood pressure of ≥90 mm Hg, the use of antihypertensive medication, or a patient-reported history of hypertension. Diabetes mellitus is identified by fasting plasma glucose levels of ≥126 mg/dl, the use of antidiabetic medication, a history of diabetes, or glycosylated haemoglobin (HbA1c) levels of ≥6.5%. Dyslipidaemia is diagnosed by total cholesterol (TC) levels of ≥240 mg/dl, triglycerides (TG) levels of ≥150 mg/dl, low-density lipoprotein cholesterol (LDL-C) levels of ≥160 mg/dl, high-density lipoprotein cholesterol (HDL-C) levels of <40 mg/dl, the use of lipid-lowering medication, or a self-reported history of dyslipidaemia [31].

### Outcome ascertainment and follow-up

In this study, the primary outcome was the incidence of CVD events. CVD was defined as a physician-diagnosed heart condition, which includes angina, heart attack, congestive heart failure, and other heart-related issues, as well as stroke [32]. The secondary outcomes were the incidence of heart problems and stroke, which were analysed separately. The date of CVD diagnosis was recorded as the time between the last interview and the interview where the CVD event was reported [3,33].

### Statistical analysis

Continuous variables that follow a Gaussian distribution are expressed as the mean ± standard deviation, while those that do not follow a Gaussian distribution are presented as the median (interquartile range (IQR)). Categorical variables are reported as frequencies (percentage). Continuous data were compared using analysis of variance (ANOVA) or the Mann-Whitney U test, and categorical data were analysed using the χ^2^ test or the Kruskal-Wallis test. Kaplan-Meier curves and the log-rank test were used to compare the cumulative risk of events across different groups.

First, we used the cox regression to investigate the association between frailty and sarcopenia status in 2011 and incident CVD in the following nine years. Subsequently, to further explore the impact of frailty and sarcopenia on incident CVD, we selected individuals from the above cohort who did not experience CVD between 2011 and 2015. We then used cox regression to analyse how changes in frailty or sarcopenia status influenced the risk of incident CVD in the following five years. Three models were finally estimated: Model 1 adjusted for age and gender. Model 2 adjusted for age, gender, HB, BMI, marital status, living place, hukou, educational level, smoking status, and drinking status. Model 3 adjusted for variables in Model 2 and the history of hypertension, diabetes, dyslipidaemia, and kidney diseases.

To evaluate the degree of mediation by frailty in the relationship between sarcopenia status and CVD, we did causal mediator analyses. We used a four-way decomposition method to account for mediation of sarcopenia and frailty on CVD with the ‘CMAverse’ R package. More details about the causal mediation analysis had been described in previous research [34–36].

All analyses were conducted using SPSS software, version 29.0 (IBM Corp., Armonk, New York, USA) and the *R* programming language, version 4.4.1 (R Foundation for Statistical Computing, Vienna, Austria). Multiple imputation was performed with the “mice” package in R, and mediation analysis was conducted using the ‘CMAverse’ package. Statistical significance was set at a two-sided *P* < 0.05.

## RESULTS

### Basic characteristics

A total of 7187 eligible participants (mean (x̄) = 57.38 years, SD = 8.41 years; male = 49.41%) were ultimately enrolled for analysis. The baseline characteristics of participants were stratified by frailty and sarcopenia status (**Table 1**). Compared to those with robust and non-sarcopenia status, frail and sarcopenia participants were older, had a higher proportion of females and agricultural hukou, lower educational levels, fewer urban residents and married people, and lower smoking and drinking rates. They also had higher incidences of hypertension, diabetes, and kidney disease, along with higher glucose and HbA1c levels and lower HB, LDL-C and TC. Baseline characteristics of participants were also stratified by frailty status or sarcopenia status (Table S2–3 in the **Online Supplementary Document**).

**Table 1:** Baseline characteristics of the study population across frailty status combined with sarcopenia status

Variables	Overall	Robust and non-sarcopenia	Robust and sarcopenia	Pre-frail and non-sarcopenia	Pre-frail and sarcopenia	Frail and non-sarcopenia	Frail and sarcopenia	*P*-value
No. of subject	7187	3407	1009	1364	828	237	342	
Age	57.38 ± 8.41	55.32 ± 7.56	58.81 ± 8.94	57.39 ± 7.92	60.67 ± 8.87	59.77 ± 7.58	64.09 ± 8.79	<0.001
Sex, n (%)								<0.001
*Male*	3551 (49.41)	1960 (57.53)	495 (49.06)	569 (41.72)	309 (37.32)	85 (35.86)	133 (38.89)	
*Female*	3636 (50.59)	1447 (42.47)	514 (50.94)	795 (58.28)	519 (62.68)	152 (64.14)	209 (61.11)	
Marital status, n (%)								<0.001
*Married*	6512 (90.61)	3187 (93.54)	891 (88.31)	1225 (89.81)	718 (86.71)	211 (89.03)	280 (81.87)	
*Others*	675 (9.39)	220 (6.46)	118 (11.69)	139 (10.19)	110 (13.29)	26 (10.97)	62 (18.13)	
Living place, n (%)								<0.001
*Urban*	2527 (35.16)	1375 (40.36)	376 (37.26)	400 (29.33)	238 (28.74)	50 (21.10)	88 (25.73)	
*Rural*	4660 (64.84)	2032 (59.64)	633 (62.74)	964 (70.67)	590 (71.26)	187 (78.90)	254 (74.27)	
Educational levels, n (%)								<0.001
*Primary school or below*	4622 (64.31)	1824 (53.54)	684 (67.79)	964 (70.67)	657 (79.35)	194 (81.86)	299 (87.43)	
*Middle school or above*	2565 (35.69)	1583 (46.46)	325 (32.21)	400 (29.33)	171 (20.65)	43 (18.14)	43 (12.57)	
BMI, (kg/m^2^)	24.30 ± 35.99	24.53 ± 43.00	24.85 ± 52.12	24.03 ± 13.42	23.78 ± 4.27	23.68 ± 3.83	23.17 ± 3.82	0.960
Smoking status, n (%)								<0.001
*Never*	4287 (59.65)	1894 (55.59)	606 (60.06)	861 (63.12)	552 (66.67)	156 (65.82)	218 (63.74)	
*Former*	549 (7.64)	269 (7.90)	66 (6.54)	107 (7.84)	55 (6.64)	20 (8.44)	32 (9.36)	
*Now*	2351 (32.71)	1244 (36.51)	337 (33.40)	396 (29.03)	221 (26.69)	61 (25.74)	92 (26.90)	
Drinking status, n (%)								<0.001
*Never*	4256 (59.22)	1881 (55.21)	624 (61.84)	832 (61.00)	563 (68.00)	140 (59.07)	216 (63.16)	
*Ever*	2931 (40.78)	1526 (44.79)	385 (38.16)	532 (39.00)	265 (32.00)	97 (40.93)	126 (36.84)	
Hukou, n (%)								<0.001
*Agriculture*	5904 (82.15)	2680 (78.66)	809 (80.18)	1168 (85.63)	724 (87.44)	213 (89.87)	310 (90.64)	
*Others*	1283 (17.85)	727 (21.34)	200 (19.82)	196 (14.37)	104 (12.56)	24 (10.13)	32 (9.36)	
Comorbidities, n (%)								
*Hypertension*	2585 (35.97)	996 (29.23)	321 (31.81)	570 (41.79)	383 (46.26)	123 (51.90)	192 (56.14)	<0.001
*Diabetes*	1078 (15.00)	440 (12.91)	123 (12.19)	244 (17.89)	139 (16.79)	61 (25.74)	71 (20.76)	<0.001
*Dyslipidaemia*	3458 (48.11)	1634 (47.96)	492 (48.76)	679 (49.78)	378 (45.65)	125 (52.74)	150 (43.86)	0.143
*Kidney diseases*	323 (4.49)	111 (3.26)	25 (2.48)	80 (5.87)	53 (6.40)	26 (10.97)	28 (8.19)	<0.001
Laboratory values								
*HB, g/dL*	14.42 ± 2.16	14.52 ± 2.14	14.39 ± 2.09	14.33 ± 2.23	14.35 ± 2.28	14.14 ± 1.96	14.21 ± 2.03	0.003
*TG, mg/dL*	132.05 ± 106.78	130.24 ± 100.41	128.84 ± 104.94	139.44 ± 125.48	132.13 ± 106.79	129.84 ± 84.47	131.45 ± 105.80	0.129
*LDL-C, mg/dL*	116.40 ± 34.36	115.50 ± 34.21	117.25 ± 34.89	117.92 ± 35.18	117.49 ± 33.59	118.90 ± 34.86	112.37 ± 31.90	0.029
*HDL-C, mg/dL*	51.17 ± 15.02	50.95 ± 15.04	51.24 ± 15.48	51.38 ± 15.14	50.77 ± 13.66	52.63 ± 15.65	52.19 ± 15.47	0.365
*TC, mg/dL*	193.95 ± 37.80	192.89 ± 38.38	193.35 ± 37.01	196.79 ± 38.41	194.74 ± 36.37	196.92 ± 36.59	190.97 ± 35.47	0.012
*FBG, mg/dL*	108.37 ± 32.28	106.91 ± 27.20	107.09 ± 29.53	109.98 ± 36.94	110.07 ± 37.51	112.47 ± 34.59	113.23 ± 47.18	<0.001
*HbA1c, %*	5.22 ± 0.73	5.17 ± 0.65	5.21 ± 0.69	5.25 ± 0.81	5.28 ± 0.82	5.35 ± 0.83	5.33 ± 0.94	<0.001

BMI – body mass index, HB – haemoglobin, TG – triglyceride, LDL – low-density lipoprotein, HDL – high-density lipoprotein, TC – total cholesterol

### Association of baseline frailty and sarcopenia status with incident CVD

During the nine-year follow-up period, 1901 participants (26.45%) developed CVD, with 1470 (20.58%) cases involving heart diseases and 632 (8.98%) cases of stroke. Participants with frail or sarcopenia had the highest risk of CVD, heart problems and stroke (Table S4 in the **Online Supplementary Document**).

**Figure 1:**
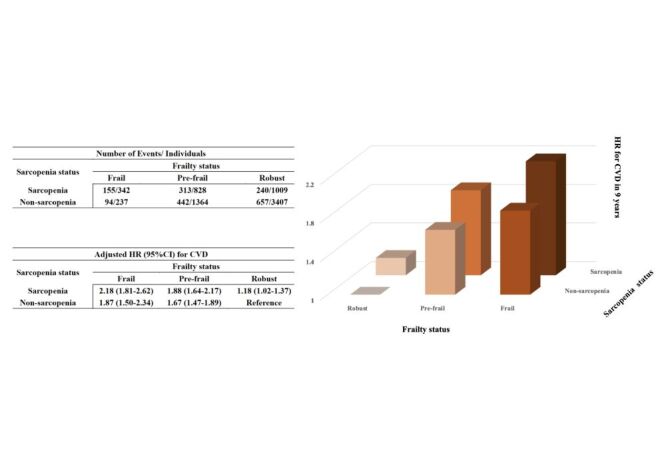
CVD risk by combined categories of frailty and sarcopenia status. Adjusted for age, gender, haemoglobin, body mass index, marital status, living place, hukou, educational level, smoking status, drinking status, and the history of hypertension, diabetes, dyslipidaemia, and kidney diseases. CVD – cardiovascular diseases; HR – hazard ratio; CI – confidence interval.

Notably, there might be a significant interaction for CVD between frailty and sarcopenia status (*P* for interaction <0.001). The Kaplan-Meier curves showed the cumulative incidence of CVD and heart problems/stroke among study participants, with all curves demonstrating statistically significant differences (log-rank test, *P* < 0.001; Figure S2–3 in the **Online Supplementary Document**). After adjusting for potential confounders, compared to participants who were robust without sarcopenia, those with combinations of frailty and sarcopenia exhibited progressively higher risks of incident CVD. Specifically, risk increased by 18% in robust individuals with sarcopenia, 67% in pre-frail without sarcopenia, 88% in pre-frail with sarcopenia, 87% in frail without sarcopenia, and 118% in frail with sarcopenia (**Figure 1**; **Table 2**). Comparable findings were observed for heart problems and stroke, except for the people with robust and sarcopenia had not a significantly increased risk of stroke (Table S5 in the **Online Supplementary Document**). This finding suggests that older adults with frailty and reduced muscle mass should be considered a very high-risk population in clinical practice, warranting prioritised comprehensive interventions. It underscores the importance of preserving physical strength in older individuals as a key strategy for primary prevention of cardiovascular disease.

**Table 2:** Association of frail status combined with sarcopenia status and CVD

Variables	Case	Model 1*		Model 2†		Model 3‡	
		**HR (95% CI)**	***P*-value**	**HR (95% CI)**	***P*-value**	**HR (95% CI)**	***P*-value**
Robust and non-sarcopenia	657/3407	Ref	Ref	Ref	Ref	Ref	Ref
Robust and sarcopenia	240/1009	1.15 (0.99–1.33)	0.072	1.17 (1.01–1.36)	0.037	1.18 (1.02–1.37)	0.030
Pre-frail and non-sarcopenia	442/1364	1.67 (1.48–1.89)	<0.001	1.77 (1.57–2.01)	<0.001	1.67 (1.47–1.89)	<0.001
Pre-frail and sarcopenia	313/828	1.86 (1.62–2.14)	<0.001	2.00 (1.74–2.30)	<0.001	1.88 (1.64–2.17)	<0.001
Frail and non-sarcopenia	94/237	1.94 (1.56–2.42)	<0.001	2.12 (1.70–2.64)	<0.001	1.87 (1.50–2.34)	<0.001
Frail and sarcopenia	155/342	2.15 (1.79–2.58)	<0.001	2.36 (1.96–2.84)	<0.001	2.18 (1.81–2.62)	<0.001

HR – hazard ratio, CI – confidence interval

*Model 1 adjusted for age and gender.

†Model 2 adjusted for age, gender, haemoglobin, body mass index, marital status, living place, hukou, educational level, smoking status, and drinking status.

‡Model 3 adjusted for variables in Model 2 and the history of hypertension, diabetes, dyslipidaemia, and kidney diseases.

### Mediation analysis

**Figure 2:**
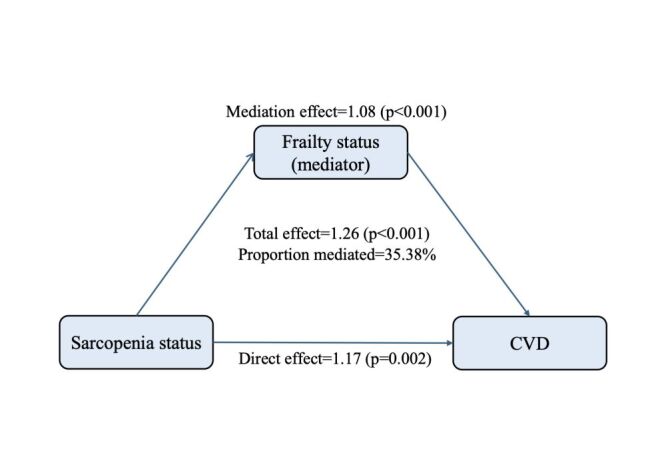
Effect of frailty status (mediator) on the relationship between sarcopenia status (exposure) and CVD (outcome) risk. Adjusted for age, gender, haemoglobin, body mass index, marital status, living place, hukou, educational level, smoking status, drinking status, and the history of hypertension, diabetes, dyslipidaemia, and kidney diseases. CVD – cardiovascular diseases.

Mediation analysis was conducted to quantify the extent to which the frailty status served as a mediator in the relationship between sarcopenia status and CVD. The mediation analysis revealed that sarcopenia status had a significant direct effect on CVD, and the frailty status partly mediated the indirect effect of sarcopenia on CVD (Table S6 in the **Online Supplementary Document**). After adjusting for confounders, frailty status was found to partially mediate the association between sarcopenia and cardiovascular outcomes (*P* < 0.001), accounting for 35.38% of the total effect on CVD (**Figure 2**). Similar mediating effects were observed for heart problems (38.66%) and stroke (27.57%) (Table S6 in the **Online Supplementary Document**).

### Association of dynamic changes in frailty status and sarcopenia status with incident CVD

**Figure 3:**
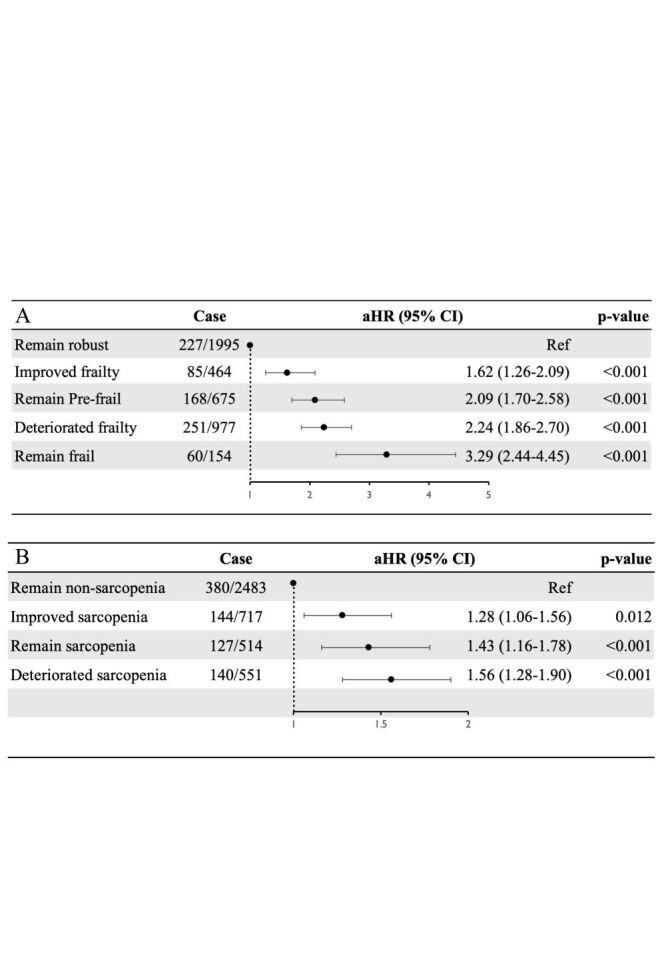
Association of the frail status change (**Panel A**) and the sarcopenia status change (**Panel B**) and CVD. Adjusted for age, gender, haemoglobin, body mass index, marital status, living place, hukou, educational level, smoking status, drinking status and the history of hypertension, diabetes, dyslipidaemia, and kidney diseases. CVD – cardiovascular diseases; HR – hazard ratio; CI – confidence interval.

Dynamic changes in frailty or sarcopenia status were associated with the risk of incident CVD among participants from 2011 to 2015 (Table S7 in the **Online Supplementary Document**). During the five years follow-up, among 4265 participants, 791 (18.55%) developed CVD, including 573 (13.48%) cases of heart problems and 279 (6.59%) cases of stroke. After adjusting for all confounders, compared to those who remained robust, participants who remained frail throughout the five years had the highest risk of incident CVD (adjusted hazard ratio (aHR) = 3.29; 95% confidence interval (CI) = 2.44–4.45) and improved frailty (aHR = 1.62; 95% CI = 1.26–2.09) had the lowest risk of incident CVD (**Figure 3**, Panel A). Compared to participants who never had sarcopenia, those with deteriorated sarcopenia had the highest risk of incident CVD (aHR = 1.56; 95% CI = 1.28–1.90) and improved sarcopenia (aHR = 1.28; 95% CI = 1.06–1.56) (**Figure 3**, Panel B). The risk of both cardiac and stroke was also highest in participants who remained frail and those deteriorated sarcopenia (Table S8–9 in the **Online Supplementary Document**).

### Sensitive analysis

In the sensitivity analysis, we evaluated the influence of demographic factors on changes in frailty and sarcopenia status. The Multivariable multinomial logistic regression results showed that older age, female, rural residence, and lower educational levels were significantly associated with adverse transitions (*e.g*. deterioration or persistence) in both frailty and sarcopenia. In contrast, higher education level was associated with better outcomes, including improvement or maintaining a robust/non-sarcopenic state. Additionally, non-agricultural hukou showed a protective effect in some transitions (Table S10–11 in the **Online Supplementary Document**). Overall, demographic characteristics play an important role in the dynamic changes of frailty and sarcopenia and should be considered when designing intervention strategies.

To verify the robustness of our findings, we conducted a sensitivity analysis excluding individuals with possible sarcopenia, comparing only those with no sarcopenia and sarcopenia. The original analysis identified the frail and sarcopenia group as having the highest CVD risk, whereas in the sensitivity analysis, after sample size adjustment, the pre-frail and sarcopenia group emerged as the highest-risk group. This shift may be due to reduced sample sizes in some subgroups affecting the stability of the estimates. The trajectory analysis of changes in sarcopenia and frailty status yielded consistent results with stable statistical conclusions (Table S12–14 in the **Online Supplementary Document**). This sensitivity analysis further supports a multidimensional understanding of the impact of sarcopenia and frailty on cardiovascular risk.

## DISCUSSION

Our study presents a complete chain, demonstrating that frailty and sarcopenia, as important risk factors, affect the risk of incident CVD in populations above 45 years old, whether independently, jointly, or through changes. To our knowledge, this research is first to report the combined and progressive effects of frailty and sarcopenia on the development of CVD. In this research, we found that frailty or sarcopenia status independently increased the risk of incident CVD. Moreover, the coexistence of frailty and sarcopenia was related to an even greater risk of CVD compared to individuals with either frailty or sarcopenia. Another finding of this research was that frailty mediated the impact of sarcopenia status on CVD. Additionally, our study revealed that dynamic changes in frailty or sarcopenia were also associated with CVD.

Our findings suggest that both frailty and sarcopenia are independently positively associated with the risk of incident CVD in populations above 45 years old. Research involving 314 093 participants from the UK Biobank reported an association between physical frailty and the incidence of CVD [11]. Another study involving 15 137 older adults in China reported that both possible sarcopenia and sarcopenia were associated with higher CVD risks [12]. Although frailty and sarcopenia have traditionally been studied as independent risk factors, the significantly increased joint effect observed in our study suggests a potential synergistic interaction between them.

This study not only considered baseline frailty and sarcopenia but also examined the link between their changes and CVD. Previous studies have separately explored the association between frailty and sarcopenia with the risk of CVD. He et al. found that CVD risk increases with frailty progression and decreases with recovery from frailty [26]. Another study considering both cognitive and frailty changes found that individuals with the worst baseline status and concurrent deterioration in both areas had the highest risk of mortality [37]. Luo et al. demonstrated that sarcopenia states in older adults exhibited bidirectional transitions and that a significant proportion of possible sarcopenia cases can recover naturally [19]. Frailty and sarcopenia, as reversible conditions, suggest that restoring physical function and muscle strength should be important intervention targets for older individuals [19,26]. These findings emphasise that frailty and sarcopenia are not static conditions, but dynamic processes influenced by biological, behavioural, and environmental factors. Their reversibility offers hope for public health interventions.

An important finding of our study is that the coexistence of frailty and sarcopenia significantly increases the risk of CVD, with frailty mediating approximately 35.38% of the association between sarcopenia and CVD. Sarcopenia is a syndrome characterised by the progressive loss of skeletal muscle mass and function, while frailty is a broader syndrome reflecting diminished physiological reserve and multisystem dysfunction [38,39]. These two conditions are closely related and often coexist [40,41]. The core pathological mechanism of sarcopenia involves chronic low-grade inflammation, where inflammatory cytokines accelerate muscle protein breakdown and inhibit muscle regeneration [42]. This inflammation-driven muscle degradation markedly reduces physical capacity and resilience, becoming a key driver of frailty development [43,44]. Frailty further exacerbates systemic inflammation and oxidative stress, leading to metabolic abnormalities, impaired immune function, and weakened stress response. These pathological changes diminish the body’s ability to maintain homeostasis, making it more susceptible to cardiovascular events [45]. Additionally, sarcopenia-induced declines in muscle strength and endurance further reduce physical activity and increase sedentary behaviour, which aggravate frailty and promote cardiovascular risk factors such as insulin resistance and endothelial dysfunction [18]. However, the complex biological and behavioural mechanisms linking these conditions require further prospective and mechanistic studies to clarify causal pathways and guide effective clinical interventions.

Our findings carry several practical implications. First, we propose integrating routine frailty and sarcopenia screening into cardiovascular risk assessment, especially in older adults. In low-resource primary care settings, this may be achieved using simple tools such as the Rockwood frailty index and anthropometry-based sarcopenia risk scores, which do not require expensive equipment. Although anthropometric estimates are less precise than DXA, they remain feasible and scalable for community-level screening and have been validated in Chinese populations [46]. Second, targeted interventions – such as structured resistance training programmes, nutritional supplementation (*e.g*. protein or vitamin D), and medication review – should be prioritised for individuals with identified frailty or sarcopenia [47]. Third, given the reversible nature of these conditions, longitudinal monitoring and stepped interventions may help improve physical function and reduce cardiovascular risk. Future studies should evaluate the cost-effectiveness and implementation feasibility of such screening protocols in real-world clinical settings [48].

This research had several limitations. First, the diagnosis of CVD in this study was based on self-reported medical diagnoses, which may be subject to recall bias and misclassification, potentially affecting outcome validity [49]. Second, sarcopenia was defined by estimating muscle mass using anthropometric equations rather than the gold-standard methods such as DXA or bioelectrical impedance analysis recommended by AWGS 2019. Although these equations have been previously validated in population studies [50], this indirect estimation may introduce measurement error. These factors, combined with the observational nature of our data, mean that unmeasured confounding cannot be completely ruled out, which should be considered in the future. Furthermore, we acknowledge that since the study population consists solely of older Chinese adults, the external validity of our findings to other ethnic groups and settings may be limited. Further validation in diverse populations is needed in the future. Although dietary habits and genetic factors were not directly measured, we adjusted for proxy variables such as BMI and comorbidity burden to reduce residual confounding. Residual confounding may remain.

## CONCLUSIONS

This research demonstrated that frailty and sarcopenia status have a significantly and independently combined and progressive impact on incident CVD in the middle-aged and older population. In future, we recommend to regular screening for frailty and sarcopenia, along with comprehensive interventions, may help slow their progression or promote recovery, thereby reducing the risk of CVD. In the process of ageing population, these findings offer new directions and strategies for improving the quality of life for older adults and reducing the burden of CVD.

## Additional material


Online Supplementary Document
