# Supplementary material for: Combined and progressive effect of frailty and sarcopenia on the risk of cardiovascular disease in middle-aged and older adults
Source: J Glob Health. 2025 Oct 31;15:04299. doi: 10.7189/jogh.15.04299 (PMC12576861; doi:10.7189/jogh.15.04299)

Supplement to: Liu ML, Li JW, Yan KL, Zhang KX, Wang M, Li TY, Guo JD, Heisha N, Yang YJ, Yuan JQ, Ye YQ, Zhao XY. Combined and progressive effect of frailty and sarcopenia on the risk of cardiovascular disease in middle-aged and older adults. J Glob Health. 2025;15:04299.

File S1 Missing data handling

Checklist S1 STROBE Statement—Checklist of items that should be included in reports of cohort studies

Table S1 The 28 items used to construct the frailty index in CHARLS

Table S2 Baseline characteristics in patients stratified by frailty status

Table S3 Baseline characteristics in patients stratified by sarcopenia status

Table S4 Association of frailty or sarcopenia status and CVD, heart problems and stroke

Table S5 Association of frail status combined with sarcopenia status and heart problems and stroke

Table S6 Mediated effects by frailty status on the associations of sarcopenia status with risk of CVD, heart problems and stroke

Table S7 Association of the frail status or sarcopenia status change and CVD

Table S8 Association of the frail status change and heart problems and stroke

Table S9 Association of the sarcopenia status change and heart problems and stroke

Table S10 Multivariable multinomial logistic regression of demographic factors associated with frailty status change

Table S11 Multivariable multinomial logistic regression of demographic factors associated with sarcopenia status change

Table S12 Association of frail status combined with sarcopenia status and CVD, heart problems and stroke (excluded possible sarcopenia)

Table S13 Association of the frail status change and CVD, heart problems and stroke (excluded possible sarcopenia)

Table S14 Association of the sarcopenia status change and CVD, heart problems and stroke(excluded possible sarcopenia)

Figure S1 Flowchart of study population selection

Figure S2 Kaplan-Meier analysis for CVD according to frailty and sarcopenia status

Figure S3 Kaplan-Meier analysis for heart problems (A) or stroke (B) according to frailty and sarcopenia status

File S1 Missing data handling

To address missing data, we first excluded participants with missing values on key variables such as sarcopenia and frailty at baseline (2011), prevalent CVD, or age below 45 years. For the remaining covariates—including hemoglobin concentration, lipid profiles, fasting glucose, HbA1c, history of dyslipidemia, kidney disease, hukou, use of antidiabetic and lipid-lowering medications, drinking history, and systolic and diastolic blood pressure—we applied the "mice" package in R with random forest imputation to fill in missing values.

In the longitudinal analysis assessing changes between 2011 and 2015 in relation to incident CVD in 2020, we further excluded individuals who developed CVD between 2011 and 2015 or had missing frailty or sarcopenia data during that period, as well as those with missing follow-up information. Covariates in this analysis were imputed using the same random forest method described above.

Checklist S1 STROBE Statement—Checklist of items that should be included in reports of cohort studies

	Item No	Recommendation	Page No
**Title and abstract**	1	(*a*) Indicate the study's design with a commonly used term in the title or the abstract	1-2
		(*b*) Provide in the abstract an informative and balanced summary of what was done and what was found	
**Introduction**			
Background/rationale	2	Explain the scientific background and rationale for the investigation being reported	2-3
Objectives	3	State specific objectives, including any prespecified hypotheses	3
**Methods**			
Study design	4	Present key elements of study design early in the paper	4
Setting	5	Describe the setting, locations, and relevant dates, including periods of recruitment, exposure, follow-up, and data collection	5-7
Participants	6	(*a*) Give the eligibility criteria, and the sources and methods of selection of participants. Describe methods of follow-up	4
		(*b*) For matched studies, give matching criteria and number of exposed	

		and unexposed	
Variables	7	Clearly define all outcomes, exposures, predictors, potential confounders, and effect modifiers. Give diagnostic criteria, if applicable	7
Data sources/ measurement	8*	For each variable of interest, give sources of data and details of methods of assessment (measurement). Describe comparability of assessment methods if there is more than one group	6-7
Bias	9	Describe any efforts to address potential sources of bias	6-7
Study size	10	Explain how the study size was arrived at	4
Quantitative variables	11	Explain how quantitative variables were handled in the analyses. If applicable, describe which groupings were chosen and why	4-7
Statistical methods	12	(*a*) Describe all statistical methods, including those used to control for confounding<br>(*b*) Describe any methods used to examine subgroups and interactions<br>(*c*) Explain how missing data were addressed<br>(*d*) If applicable, explain how loss to follow-up was addressed<br>(*e*) Describe any sensitivity analyses	6-7

Results			
Participants	13*	(a) Report numbers of individuals at each stage of study—eg numbers potentially eligible, examined for eligibility, confirmed eligible, included in the study, completing follow-up, and analysed<br><br>(b) Give reasons for non-participation at each stage<br><br>(c) Consider use of a flow diagram	7-8
Descriptive data	14*	(a) Give characteristics of study participants (eg demographic, clinical, social) and information on exposures and potential confounders<br><br>(b) Indicate number of participants with missing data for each variable of interest<br><br>(c) Summarise follow-up time (eg, average and total amount)	8-9
Outcome data	15*	Report numbers of outcome events or summary measures over time	7-9

Main results	16	(*a*) Give unadjusted estimates and, if applicable, confounder-adjusted estimates and their precision (eg, 95% confidence interval). Make clear which confounders were adjusted for and why they were included<br><br>(*b*) Report category boundaries when continuous variables were categorized<br><br>(*c*) If relevant, consider translating estimates of relative risk into absolute risk for a meaningful time period	7-9
Other analyses	17	Report other analyses done—eg analyses of subgroups and interactions, and sensitivity analyses	8-9
**Discussion**			
Key results	18	Summarise key results with reference to study objectives	10
Limitations	19	Discuss limitations of the study, taking into account sources of potential bias or imprecision. Discuss both direction and magnitude of any potential bias	12-13
Interpretation	20	Give a cautious overall interpretation of results considering objectives, limitations, multiplicity of analyses, results from similar studies, and other relevant evidence	11-13
Generalisability	21	Discuss the generalisability (external validity) of the study results	12-13
**Other information**			
Funding	22	Give the source of funding and the role of the funders for the present study and, if	13

applicable, for the original study on which the present article is based	

*Give information separately for exposed and unexposed groups.

Note: An Explanation and Elaboration article discusses each checklist item and gives methodological background and published examples of transparent reporting. The STROBE checklist is best used in conjunction with this article (freely available on the Web sites of PLoS Medicine at http://www.plosmedicine.org/, Annals of Internal Medicine at http://www.annals.org/, and Epidemiology at http://www.epidem.com/). Information on the STROBE Initiative is available at http://www.strobe-statement.org.

Table S1 The 28 items used to construct the frailty index in CHARLS

No	Description of the item	Cut-off value
1	Self-reported physician diagnosed hypertension	Yes = 1, No = 0
2	Self-reported physician diagnosed diabetes	Yes = 1, No = 0
3	Self-reported physician diagnosed cancer	Yes = 1, No = 0
4	Self-reported physician diagnosed arthritis	Yes = 1, No = 0
5	Self-reported physician diagnosed chronic lung disease	Yes = 1, No = 0
6	Self-reported physician diagnosed any emotional, nervous, or psychiatric problems	Yes = 1, No = 0
7	Self-reported physician diagnosed memory-related disease	Yes = 1, No = 0
8	Self-reported vision problems	Yes = 1, No = 0
9	Self-reported hearing problems	Yes = 1, No = 0
10	Self-reported general health status	Very poor or poor = 1, Very good, good, or fair = 0
11	Difficulty with dressing	Yes = 1, No = 0
12	Difficulty with bathing or showering	Yes = 1, No = 0
13	Difficulty with eating	Yes = 1, No = 0
14	Difficulty with getting in and out of bed	Yes = 1, No = 0
15	Difficulty with using the toilet	Yes = 1, No = 0
16	Difficulty with managing money	Yes = 1, No = 0
17	Difficulty with taking medication	Yes = 1, No = 0
18	Difficulty with shopping for groceries	Yes = 1, No = 0
19	Difficulty with preparing meals	Yes = 1, No = 0
20	Mobility: difficulty with walking 100 yards or one block	Yes = 1, No = 0
21	Mobility: difficulty with getting up from a chair after sitting for long periods	Yes = 1, No = 0

22	Mobility: difficulty with climbing several flights of stairs without resting	Yes = 1, No = 0
23	Mobility: difficulty with lifting or carrying weights over 10 pounds/jins	Yes = 1, No = 0
24	Mobility: difficulty with picking up a coin from the table	Yes = 1, No = 0
25	Mobility: difficulty with stooping, kneeling, or crouching	Yes = 1, No = 0
26	Mobility: difficulty with reaching arms above shoulder level	Yes = 1, No = 0
27	Depression: CESD-10 questionnaire	CESD-10 $>10 = 1$, $\leq 10 = 0$
28	Cognition: (memory test score + orientation test score) / 14	Continuous variable, ranging from 0 to 1

Depression was assessed by the Center for Epidemiologic Studies Depression Scale (CESD). In the CHARLS, CESD-10 was used, and the total score ranged from 0 to 30. A higher score indicated more severe depressive symptoms.

The memory score was the average of words which were not recalled in the immediate and delayed word recall tasks. The memory score ranged from 0 to 10. The orientation test comprised 4 questions about the day of the week, the month, the date of the month, and the year. One point was given for each wrong answer, and the range was from 0 to 4.

Table S2 Baseline characteristics in patients stratified by frailty status

Variables	Total population (n=7,187)	Robust (n=4,416)	Pre-frail (n=2,192)	Frail (n=579)	p-value
**Demographics**					
Age, years	57.38±8.41	56.12±8.03	58.62±8.44	62.32±8.58	<0.001
Sex, n (%)					<0.001
Male	3551 (49.41)	2455 (55.59)	878 (40.05)	218 (37.65)	
Female	3636 (50.59)	1961 (44.41)	1314 (59.95)	361 (62.35)	
Marital status, n (%)					<0.001
Married	6512 (90.61)	4078 (92.35)	1943 (88.64)	491 (84.80)	
Others	675 (9.39)	338 (7.65)	249 (11.36)	88 (15.20)	
Living place, n (%)					<0.001
Urban	2527 (35.16)	1751 (39.65)	638 (29.11)	138 (23.83)	
Rural	4660 (64.84)	2665 (60.35)	1554 (70.89)	441 (76.17)	
Educational levels, n (%)					<0.001
Primary school or below	4622 (64.31)	2508 (56.79)	1621 (73.95)	493 (85.15)	
Middle school or above	2565 (35.69)	1908 (43.21)	571 (26.05)	86 (14.85)	
BMI, (kg/m^2)	24.30±35.99	24.60±45.24	23.94±10.91	23.38±3.83	0.634
Smoking status, n (%)					<0.001
Never	4287 (59.65)	2500 (56.61)	1413 (64.46)	374 (64.59)	

Former	549 (7.64)	335 (7.59)	162 (7.39)	52 (8.98)	
Now	2351 (32.71)	1581 (35.80)	617 (28.15)	153 (26.42)	
Drinking status, n (%)					<0.001
Never	4256 (59.22)	2505 (56.73)	1395 (63.64)	356 (61.49)	
ever	2931 (40.78)	1911 (43.27)	797 (36.36)	223 (38.51)	
Hukou, n (%)					<0.001
agriculture	5904 (82.15)	3489 (79.01)	1892 (86.31)	523 (90.33)	
others	1283 (17.85)	927 (20.99)	300 (13.69)	56 (9.67)	
**Comorbidities**					
Hypertension, n (%)	2585 (35.97)	1317 (29.82)	953 (43.48)	315 (54.40)	<0.001
Diabetes, n (%)	1078 (15.00)	563 (12.75)	383 (17.47)	132 (22.80)	<0.001
Dyslipidemia, n (%)	3458 (48.11)	2126 (48.14)	1057 (48.22)	275 (47.50)	0.951
Kidney diseases, n (%)	323 (4.49)	136 (3.08)	133 (6.07)	54 (9.33)	<0.001
**Laboratory values**					
HB, g/dL	14.42±2.16	14.49±2.13	14.34±2.25	14.19±2.00	0.001
TG, mg/dL	132.05±106.78	129.92±101.45	136.68±118.79	130.79±97.56	0.051
LDL-C, mg/dL	116.40±34.36	115.90±34.37	117.76±34.58	115.04±33.27	0.072
HDL-C, mg/dL	51.17±15.02	51.02±15.15	51.15±14.60)	52.37±15.53	0.124
TC, mg/dL	193.95±37.80	193.00±38.07	196.02±37.66)	193.41±36.02	0.009
FBG, mg/dL	108.37±32.28	106.95±27.74	110.01±37.14	112.92±42.44	<0.001
HbA1c, %	5.22±0.73	5.18±0.66	5.26±0.81	5.34±0.89	<0.001

BMI: body mass index; TG: triglyceride; LDL, low-density lipoprotein; TC, total cholesterol; HDL, high-density lipoprotein.

Table S3 Baseline characteristics in patients stratified by sarcopenia status

Variables	Total population (n=7,187)	Non-sarcopenia (n=5,008)	Sarcopenia (n=2,179)	p-value
**Demographics**				
Age, years	57.38±8.41	56.09±7.76	60.34±9.07	<0.001
Sex, n (%)				<0.001
Male	3551 (49.41)	2614 (52.20)	937 (43.00)	
Female	3636 (50.59)	2394 (47.80)	1242 (57.00)	
Marital status, n (%)				<0.001
Married	6512 (90.61)	4623 (92.31)	1889 (86.69)	
Others	675 (9.39)	385 (7.69)	290 (13.31)	
Living place, n (%)				<0.001
Urban	2527 (35.16)	1825 (36.44)	702 (32.22)	
Rural	4660 (64.84)	3183 (63.56)	1477 (67.78)	
Educational levels, n (%)				<0.001
Primary school or below	4622 (64.31)	2982 (59.54)	1640 (75.26)	
Middle school or above	2565 (35.69)	2026 (40.46)	539 (24.74)	
BMI, (kg/m^2)	24.30±35.99	24.35±36.16	24.18±35.59	0.854
Smoking status, n (%)				<0.001
Never	4287 (59.65)	2911 (58.13)	1376 (63.15)	

Former	549 (7.64)	396 (7.91)	153 (7.02)	
Now	2351 (32.71)	1701 (33.97)	650 (29.83)	
Drinking status, n (%)				<0.001
Never	4256 (59.22)	2853 (56.97)	1403 (64.39)	
ever	2931 (40.78)	2155 (43.03)	776 (35.61)	
Hukou, n (%)				<0.001
agriculture	5904 (82.15)	4061 (81.09)	1843 (84.58)	
others	1283 (17.85)	947 (18.91)	336 (15.42)	
**Comorbidities**				
Hypertension, n (%)	2585 (35.97)	1689 (33.73)	896 (41.12)	<0.001
Diabetes, n (%)	1078 (15.00)	745 (14.88)	333 (15.28)	0.684
Dyslipidemia, n (%)	3458 (48.11)	2438 (48.68)	1020 (46.81)	0.152
Kidney diseases, n (%)	323 (4.49)	217 (4.33)	106 (4.86)	0.348
**Laboratory values**				
HB, g/dL	14.42±2.16	14.45±2.16	14.35±2.15	0.061
TG, mg/dL	132.05±106.78	132.73±107.23	130.50±105.74	0.416
LDL-C, mg/dL	116.40±34.36	116.32±34.52	116.58±33.98	0.770
HDL-C, mg/dL	51.17±15.02	51.15±15.10	51.21±14.82	0.862
TC, mg/dL	193.95 (37.80)	194.15±38.35	193.51±36.53	0.509
FBG, mg/dL	108.37±32.28	108.01±30.56	109.19±35.93	0.156
HbA1c, %	5.22±0.73	5.20±0.71	5.25±0.78	0.003

BMI: body mass index; TG: triglyceride; LDL, low-density lipoprotein; TC, total cholesterol; HDL, high-density lipoprotein.

Table S4 Association of frailty or sarcopenia status and CVD, heart problems and stroke

	case	Model 1		Model 2		Model 3	
		aHR (95% CI)	p-value	aHR (95% CI)	p-value	aHR (95% CI)	p-value
**CVD**							
**Frailty status**	1901/7187						
Robust	897/4416	Ref	Ref	Ref	Ref	Ref	Ref
Pre-frail	755/2192	1.68 (1.52-1.85)	<0.001	1.78 (1.61-1.96)	<0.001	1.67 (1.51-1.85)	<0.001
Frail	249/579	1.98 (1.71-2.29)	<0.001	2.15 (1.85-2.49)	<0.001	1.94 (1.67-2.26)	<0.001
**Sarcopenia status**	1901/7187						
Non-sarcopenia	1193/5008	Ref	Ref	Ref	Ref	Ref	Ref
Sarcopenia	708/2179	1.24 (1.13-1.37)	<0.001	1.27 (1.15-1.40)	<0.001	1.26 (1.14-1.39)	<0.001
**Heart problems**							
**Frailty status**	1470/7142						
Robust	672/4397	Ref	Ref	Ref	Ref	Ref	Ref
Pre-frail	608/2176	1.76 (1.57-1.97)	<0.001	1.89 (1.69-2.12)	<0.001	1.79 (1.59-2.00)	<0.001
Frail	190/569	1.95 (1.65-2.30)	<0.001	2.17 (1.83-2.57)	<0.001	1.99 (1.67-2.36)	<0.001
**Sarcopenia status**	1470/7142						
Non-sarcopenia	919/4982	Ref	Ref	Ref	Ref	Ref	Ref
Sarcopenia	551/2160	1.23 (1.10-1.38)	<0.001	1.27 (1.14-1.42)	<0.001	1.26 (1.13-1.41)	<0.001
**Stroke**							
**Frailty status**	632/7041						
Robust	298/4362	Ref	Ref	Ref	Ref	Ref	Ref
Pre-frail	235/2126	1.58 (1.32-1.88)	<0.001	1.60 (1.34-1.91)	<0.001	1.45 (1.21-1.73)	<0.001
Frail	99/553	2.38 (1.88-3.02)	<0.001	2.44 (1.92-3.10)	<0.001	2.10 (1.65-2.68)	<0.001
**Sarcopenia status**	632/7041						

Non-sarcopenia	392/4941	Ref	Ref	Ref	Ref	Ref	Ref
Sarcopenia	240/2100	1.27 (1.08-1.50)	0.005	1.27 (1.07-1.50)	0.005	1.26 (1.07-1.49)	0.006

Model 1 adjusted for age and gender.

Model 2 adjusted for age, gender, HB, BMI, marital status, living place, hukou, educational level, smoking status, and drinking status.

Model 3 adjusted for variables in Model 2 and the history of hypertension, diabetes, dyslipidemia, and kidney diseases.

Table S5 Association of frail status combined with sarcopenia status and heart problems and stroke

		Model 1		Model 2		Model 3	
	Case	HR	p-value	HR	p-value	HR	p-value
**Heart problems**							
Robust and non-sarcopenia	488/3394	Ref	Ref	Ref	Ref	Ref	Ref
Robust and sarcopenia	184/1003	1.17 (0.99-1.39)	0.069	1.21 (1.02-1.44)	0.031	1.22 (1.02-1.44)	0.026
Pre-frail and non-sarcopenia	357/1356	1.78 (1.55-2.04)	<0.001	1.91 (1.66-2.20)	<0.001	1.80 (1.57-2.08)	<0.001
Pre-frail and sarcopenia	251/820	1.94 (1.66-2.28)	<0.001	2.13 (1.82-2.50)	<0.001	2.02 (1.72-2.37)	<0.001
Frail and non-sarcopenia	74/232	2.01 (1.57-2.57)	<0.001	2.24 (1.75-2.88)	<0.001	2.02 (1.57-2.60)	<0.001
Frail and sarcopenia	116/337	2.08 (1.68-2.56)	<0.001	2.35 (1.90-2.91)	<0.001	2.18 (1.75-2.70)	<0.001
**Stroke**							
Robust and non-sarcopenia	223/3375	Ref	Ref	Ref	Ref	Ref	Ref
Robust and sarcopenia	75/987	1.06 (0.81-1.38)	0.666	1.07 (0.82-1.39)	0.637	1.07 (0.82-1.40)	0.606
Pre-frail and non-sarcopenia	135/1336	1.51 (1.22-1.88)	<0.001	1.53 (1.23-1.90)	<0.001	1.38 (1.11-1.72)	0.004
Pre-frail and sarcopenia	100/790	1.75 (1.37-2.24)	<0.001	1.79 (1.39-2.28)	<0.001	1.64 (1.28-2.10)	<0.001
Frail and non-sarcopenia	34/230	2.15 (1.50-3.10)	<0.001	2.23 (1.54-3.22)	<0.001	1.85 (1.28-2.68)	0.001
Frail and sarcopenia	65/323	2.62 (1.96-3.50)	<0.001	2.68 (1.99-3.59)	<0.001	2.37 (1.76-3.18)	<0.001

Model 1 adjusted for age and gender.

Model 2 adjusted for age, gender, HB, BMI, marital status, living place, hukou, educational level, smoking status, and drinking status.

Model 3 adjusted for variables in Model 2 and the history of hypertension, diabetes, dyslipidemia, and kidney diseases.

Table S6 Mediated effects by frailty status on the associations of sarcopenia status with risk of heart problems and stroke

Model	Total effect (TE)		Total natural direct effect (TNDE)		Total natural indirect effect (TNIE)		Proportion of effect mediated
	HR (95% CI)	p value	HR (95% CI)	p value	HR (95% CI)	p value	
**CVD**							
Model 1	1.23 (1.12-1.37)	p<0.001	1.13 (1.03-1.25)	p=0.010	1.09 (1.07-1.12)	p<0.001	43.17%
Model 2	1.26 (1.14-1.39)	p<0.001	1.15 (1.04-1.27)	p<0.001	1.09 (1.07-1.12)	p<0.001	40.70%
Model 3	1.26 (1.15-1.40)	p<0.001	1.17 (1.07-1.28)	p=0.002	1.08 (1.06-1.11)	p<0.001	35.38%
**Heart problems**							
Model 1	1.23 (1.10-1.37)	p<0.001	1.12 (1.00-1.25)	p=0.052	1.09 (1.07-1.13)	p<0.001	46.66%
Model 2	1.26 (1.14-1.41)	p<0.001	1.15 (1.03-1.28)	p=0.014	1.10 (1.08-1.13)	p<0.001	43.79%
Model 3	1.26 (1.13-1.41)	p<0.001	1.16 (1.04-1.29)	p=0.008	1.09 (1.06-1.12)	p<0.001	38.66%
**Stroke**							
Model 1	1.22 (1.03-1.42)	p=0.028	1.13 (0.96-1.33)	p=0.162	1.08 (1.04-1.11)	p<0.001	40.99%
Model 2	1.22 (1.02-1.43)	p=0.034	1.13 (0.96-1.34)	p=0.140	1.07 (1.04-1.11)	p<0.001	38.78%
Model 3	1.22 (1.03-1.44)	p=0.022	1.15 (0.98-1.37)	p=0.094	1.05 (1.03-1.09)	p<0.001	27.57%

Model 1 adjusted for age and gender.

Model 2 adjusted for age, gender, HB, BMI, marital status, living place, hukou, educational level, smoking status, and drinking status.

Model 3 adjusted for variables in Model 2 and the history of hypertension, diabetes, dyslipidemia, and kidney diseases.

Table S7 Association of the frail status or sarcopenia status change and CVD

	Case	Model 1		Model 2		Model 3	
		aHR (95% CI)	p-value	aHR (95% CI)	p-value	aHR(95% CI)	p-value
**Frailty status**							
Remain robust	227/1995	Ref	Ref	Ref	Ref	Ref	Ref
Improved frailty	85/464	1.59 (1.24-2.04)	<0.001	1.69 (1.31-2.17)	<0.001	1.62 (1.26-2.09)	<0.001
Remain pre-frail	168/675	2.15 (1.75-2.63)	<0.001	2.24 (1.82-2.75)	<0.001	2.09 (1.70-2.58)	<0.001
Deteriorated frailty	251/977	2.27 (1.90-2.73)	<0.001	2.38 (1.98-2.86)	<0.001	2.24 (1.86-2.70)	<0.001
Remain frail	60/154	3.30 (2.46-4.43)	<0.001	3.48 (2.58-4.68)	<0.001	3.29 (2.44-4.45)	<0.001
**Sarcopenia status**							
Remain non-sarcopenia	380/2483	Ref	Ref	Ref	Ref	Ref	Ref
Improved sarcopenia	144/717	1.26 (1.04-1.52)	0.021	1.26 (1.04-1.53)	0.018	1.28 (1.06-1.56)	0.012
Remain sarcopenia	127/514	1.42 (1.15-1.76)	0.001	1.46 (1.18-1.81)	<0.001	1.43 (1.16-1.78)	<0.001
Deteriorated sarcopenia	140/551	1.54 (1.27-1.88)	<0.001	1.57 (1.28-1.91)	<0.001	1.56 (1.28-1.90)	<0.001

Model 1 adjusted for age and gender.

Model 2 adjusted for age, gender, HB, BMI, marital status, living place, hukou, educational level, smoking status, and drinking status.

Model 3 adjusted for variables in Model 2 and the history of hypertension, diabetes, dyslipidemia, and kidney diseases.

Table S8 Association of the frail status change and heart problems and stroke

	Case	Model 1		Model 2		Model 3	
		aHR (95% CI)	p-value	aHR (95% CI)	p-value	aHR (95% CI)	p-value
**Heart problems**							
Remain robust	164/1992	Ref	Ref	Ref	Ref	Ref	Ref
Improved frailty	63/463	1.59 (1.19-2.13)	0.002	1.68 (1.25-2.25)	<0.001	1.64 (1.22-2.20)	0.001
Remain Pre-frail	130/672	2.19 (1.73-2.77)	<0.001	2.28 (1.80-2.89)	<0.001	2.18 (1.71-2.77)	<0.001
Deteriorated frailty	170/973	2.03 (1.63-2.52)	<0.001	2.11 (1.70-2.64)	<0.001	2.03 (1.63-2.54)	<0.001
Remain frail	46/151	3.33 (2.38-4.67)	<0.001	3.49 (2.48-4.92)	<0.001	3.41 (2.41-4.82)	<0.001
**Stroke**							
Remain robust	72/1988	Ref	Ref	Ref	Ref	Ref	Ref
Improved frailty	28/459	1.72 (1.11-2.66)	0.016	1.82 (1.17-2.84)	0.008	1.68 (1.08-2.62)	0.022
Remain Pre-frail	58/668	2.47 (1.73-3.51)	<0.001	2.59 (1.82-3.70)	<0.001	2.30 (1.61-3.30)	<0.001
Deteriorated frailty	102/973	3.05 (2.25-4.15)	<0.001	3.20 (2.35-4.37)	<0.001	2.88 (2.10-3.94)	<0.001
Remain frail	19/147	3.60 (2.14-6.04)	<0.001	3.84 (2.27-6.49)	<0.001	3.40 (2.00-5.77)	<0.001

Model 1 adjusted for age and gender.

Model 2 adjusted for age, gender, HB, BMI, marital status, living place, hukou, educational level, smoking status, and drinking status.

Model 3 adjusted for variables in Model 2 and the history of hypertension, diabetes, dyslipidemia, and kidney diseases.

Table S9 Association of the sarcopenia status change and heart problems and stroke

	Case	Model 1		Model 2		Model 3	
		aHR (95% CI)	p-value	aHR (95% CI)	p-value	aHR (95% CI)	p-value
**Heart problems**							
Remain non-sarcopenia	278/2476	Ref	Ref	Ref	Ref	Ref	Ref
Improved sarcopenia	101/715	1.18 (0.94-1.48)	0.164	1.18 (0.94-1.49)	0.149	1.20 (0.95-1.51)	0.126
Remain sarcopenia	92/512	1.37 (1.07-1.75)	0.014	1.40 (1.09-1.80)	0.008	1.38 (1.08-1.78)	0.011
Deteriorated sarcopenia	102/548	1.51 (1.20-1.90)	<0.001	1.53 (1.21-1.94)	<0.001	1.52 (1.20-1.92)	<0.001
**Stroke**							
Remain non-sarcopenia	134/2473	Ref	Ref	Ref	Ref	Ref	Ref
Improved sarcopenia	49/716	1.23 (0.88-1.71)	0.227	1.23 (0.88-1.71)	0.220	1.25 (0.90-1.74)	0.182
Remain sarcopenia	47/501	1.51 (1.06-2.14)	0.022	1.55 (1.09-2.21)	0.016	1.52 (1.07-2.17)	0.020
Deteriorated sarcopenia	49/545	1.52 (1.09-2.13)	0.014	1.54 (1.10-2.15)	0.013	1.52 (1.09-2.13)	0.015

Model 1 adjusted for age and gender.

Model 2 adjusted for age, gender, HB, BMI, marital status, living place, hukou, educational level, smoking status, and drinking status.

Model 3 adjusted for variables in Model 2 and the history of hypertension, diabetes, dyslipidemia, and kidney diseases.

Table S10 Multivariable multinomial logistic regression of demographic factors associated with frailty status change

Frailty status	Demographic factors	OR (95% CI)	p-value
Improved frailty	Age	1.03 (1.01-1.04)	<0.001
vs.	Sex (female vs. male)	1.58 (1.27-1.95)	<0.001
Remain robust	Married status (others vs. married)	1.45 (1.00-2.10)	0.049
	Living place (rural vs. urban)	1.68 (1.30-2.17)	<0.001
	Educational levels (middle school or above vs. primary school or below)	0.71 (0.56-0.90)	0.005
	Hukou (others vs. agriculture)	0.52 (0.35-0.77)	<0.001
Remain Pre-frail	Age	1.04 (1.03-1.05)	<0.001
vs.	Sex (female vs. male)	2.23 (1.85-2.70)	<0.001
Remain robust	Married status (others vs. married)	1.08 (0.77-1.52)	0.653
	Living place (rural vs. urban)	1.63 (1.31-2.02)	<0.001
	Educational levels (primary school or below vs. middle school or above)	0.55 (0.44-0.68)	<0.001
	Hukou (others vs. agriculture)	0.98 (0.74-1.31)	0.906
Deteriorated frailty	Age	1.03 (1.02-1.04)	<0.001
vs.	Sex (female vs. male)	2.03 (1.72-2.39)	<0.001
Remain robust	Married status (others vs. married)	1.43 (1.07-1.92)	0.015
	Living place (rural vs. urban)	1.41 (1.17-1.70)	<0.001
	Educational levels (middle school or above vs. primary school or below)	0.58 (0.48-0.70)	<0.001
	Hukou (others vs. agriculture)	0.74 (0.57-0.96)	0.022
Remain frail	Age	1.10 (1.07-1.12)	<0.001
vs.	Sex (female vs. male)	3.52 (2.43-5.10)	<0.001

Remain robust	Married status (others vs. married)	0.77 (0.43-1.40)	0.397
	Living place (rural vs. urban)	1.85 (1.21-2.83)	0.005
	Educational levels (middle school or above vs. primary school or below)	0.46 (0.28-0.74)	0.001
	Hukou (others vs. agriculture)	0.71 (0.39-1.32)	0.285

Table S11 Multivariable multinomial logistic regression of demographic factors associated with sarcopenia status change

Sarcopenia status	Demographic factors	OR (95% CI)	p-value
Improved sarcopenia	Age	1.04 (1.02-1.05)	<0.001
vs.	Sex (female vs. male)	1.44 (1.20-1.71)	<0.001
Remain non-sarcopenia	Married status (others vs. married)	0.98 (0.70-1.36)	0.883
	Living place (rural vs. urban)	1.05 (0.86-1.28)	0.634
	Educational levels (middle school or above vs. primary school or below)	0.68 (0.56-0.83)	<0.001
	Hukou (others vs. agriculture)	1.15 (0.89-1.49)	0.299
Remain sarcopenia	Age	1.11 (1.09-1.12)	<0.001
vs.	Sex (female vs. male)	1.54 (1.25-1.91)	<0.001
Remain non-sarcopenia	Married status (others vs. married)	1.50 (1.09-2.06)	0.013
	Living place (rural vs. urban)	1.41 (1.10-1.80)	0.007
	Educational levels (middle school or above vs. primary school or below)	0.65 (0.51-0.85)	0.001
	Hukou (others vs. agriculture)	0.65 (0.45-0.92)	0.016
Deteriorated sarcopenia	Age	1.07 (1.06-1.09)	<0.001
vs.	Sex (female vs. male)	1.33 (1.09-1.62)	0.005
Remain non-sarcopenia	Married status (others vs. married)	1.17 (0.83-1.63)	0.369
	Living place (rural vs. urban)	1.37 (1.09-1.73)	0.008
	Educational levels (middle school or above vs. primary school or below)	0.67 (0.53-0.84)	0.001
	Hukou (others vs. agriculture)	0.76 (0.55-1.06)	0.105

Table S12 Association of frail status combined with sarcopenia status and CVD, heart problems and stroke (excluded possible sarcopenia)

		Model 1		Model 2		Model 3	
	Case	HR	p-value	HR	p-value	HR	p-value
				**CVD**			
Robust and non-sarcopenia	657/3401	Ref	Ref	Ref	Ref	Ref	Ref
Robust and sarcopenia	47/220	0.88 (0.65-1.20)	0.430	0.94 (0.69-1.27)	0.663	1.01 (0.74-1.37)	0.965
Pre-frail and non-sarcopenia	442/1364	1.68 (1.49-1.90)	<0.001	1.79 (1.58-2.02)	<0.001	1.69 (1.49-1.91)	<0.001
Pre-frail and sarcopenia	71/189	1.61 (1.24-2.08)	<0.001	1.82 (1.40-2.36)	<0.001	1.91 (1.47-2.48)	<0.001
Frail and non-sarcopenia	94/237	1.95 (1.56-2.43)	<0.001	2.12 (1.70-2.65)	<0.001	1.90 (1.52-2.39)	<0.001
Frail and sarcopenia	41/113	1.46 (1.05-2.02)	0.025	1.62 (1.16-2.26)	0.005	1.64 (1.17-2.28)	0.004
				**Heart problems**			
Robust and non-sarcopenia	488/3394	Ref	Ref	Ref	Ref	Ref	Ref
Robust and sarcopenia	39/220	0.98 (0.71-1.37)	0.905	1.04 (0.74-1.46)	0.812	1.10 (0.78-1.55)	0.572
Pre-frail and non-sarcopenia	357/1356	1.80 (1.56-2.06)	<0.001	1.92 (1.67-2.21)	<0.001	1.84 (1.59-2.12)	<0.001
Pre-frail and sarcopenia	62/186	1.89 (1.43-2.49)	<0.001	2.16 (1.63-2.87)	<0.001	2.26 (1.70-3.00)	<0.001
Frail and non-sarcopenia	74/232	2.02 (1.58-2.60)	<0.001	2.25 (1.75-2.90)	<0.001	2.09 (1.62-2.69)	<0.001
Frail and sarcopenia	30/113	1.36 (0.93-2.00)	0.115	1.54 (1.05-2.27)	0.030	1.54 (1.05-2.28)	0.029
				**Stroke**			
Robust and non-sarcopenia	223/3375	Ref	Ref	Ref	Ref	Ref	Ref
Robust and sarcopenia	10/211	0.56 (0.28-1.05)	0.071	0.57 (0.30-1.08)	0.087	0.65 (0.34-1.25)	0.198
Pre-frail and non-sarcopenia	135/1336	1.53 (1.23-1.90)	<0.001	1.56 (1.25-1.95)	<0.001	1.40 (1.12-1.75)	0.003
Pre-frail and sarcopenia	15/178	0.99 (0.58-1.70)	0.972	1.06 (0.62-1.83)	0.830	1.15 (0.67-1.99)	0.614
Frail and non-sarcopenia	34/230	2.19 (1.52-3.16)	<0.001	2.28 (1.57-3.31)	<0.001	1.86 (1.28-2.71)	0.001

Frail and sarcopenia	17/109	1.82 (1.08-3.06)	0.024	1.90 (1.12-3.20)	0.017	1.93 (1.14-3.28)	0.014

Model 1 adjusted for age and gender.

Model 2 adjusted for age, gender, HB, BMI, marital status, living place, hukou, educational level, smoking status, and drinking status.

Model 3 adjusted for variables in Model 2 and the history of hypertension, diabetes, dyslipidemia, and kidney diseases.

Table S13 Association of the frail status change and CVD, heart problems and stroke (excluded possible sarcopenia)

	Case	Model 1		Model 2		Model 3	
		aHR (95% CI)	p-value	aHR (95% CI)	p-value	aHR (95% CI)	p-value
**CVD**							
Remain robust	187/1670	Ref	Ref	Ref	Ref	Ref	Ref
Improved frailty	55/324	1.49 (1.10-2.01)	0.010	1.59 (1.17-2.16)	0.003	1.54 (1.13-2.09)	0.006
Remain Pre-frail	118/493	2.08 (1.65-2.63)	<0.001	2.16 (1.70-2.74)	<0.001	2.02 (1.59-2.57)	<0.001
Deteriorated frailty	183/738	2.24 (1.82-2.75)	<0.001	2.35 (1.90-2.89)	<0.001	2.22 (1.80-2.74)	<0.001
Remain frail	39/98	3.35 (2.34-4.78)	<0.001	3.47 (2.41-4.99)	<0.001	3.32 (2.29-4.79)	<0.001
**Heart problems**							
Remain robust	133/1667	Ref	Ref	Ref	Ref	Ref	Ref
Improved frailty	47/324	1.75 (1.25-2.45)	0.001	1.85 (1.32-2.60)	<0.001	1.84 (1.30-2.57)	<0.001
Remain Pre-frail	93/491	2.23 (1.70-2.92)	<0.001	2.30 (1.75-3.02)	<0.001	2.23 (1.69-2.94)	<0.001
Deteriorated frailty	124/735	2.04 (1.59-2.62)	<0.001	2.14 (1.66-2.75)	<0.001	2.08 (1.62-2.67)	<0.001
Remain frail	32/95	3.75 (2.51-5.59)	<0.001	3.83 (2.55-5.77)	<0.001	3.85 (2.55-5.81)	<0.001
**Stroke**							
Remain robust	62/1664	Ref	Ref	Ref	Ref	Ref	Ref
Improved frailty	13/320	1.14 (0.61-2.03)	0.723	1.21 (0.66-2.22)	0.537	1.11 (0.60-2.03)	0.738
Remain Pre-frail	40/491	2.25 (1.50-3.37)	<0.001	2.36 (1.57-3.56)	<0.001	2.03 (1.34-3.08)	<0.001
Deteriorated frailty	74/736	2.85 (2.03-4.02)	<0.001	3.00 (2.11-4.25)	<0.001	2.63 (1.85-3.74)	<0.001
Remain frail	10/93	2.95 (1.48-5.85)	0.002	3.19 (1.59-6.39)	0.001	2.74 (1.36-5.54)	0.005

Model 1 adjusted for age and gender.

Model 2 adjusted for age, gender, HB, BMI, marital status, living place, hukou, educational level, smoking status, and drinking status.

Model 3 adjusted for variables in Model 2 and the history of hypertension, diabetes, dyslipidemia, and kidney diseases.

Table S14 Association of the sarcopenia status change and CVD, heart problems and stroke(excluded possible sarcopenia)

	Case	Model 1		Model 2		Model 3	
		aHR (95% CI)	p-value	aHR (95% CI)	p-value	aHR (95% CI)	p-value
**CVD**							
Remain non-sarcopenia	380/2483	Ref	Ref	Ref	Ref	Ref	Ref
Improved sarcopenia	25/139	1.00 (0.66-1.51)	0.988	1.02 (0.68-1.55)	0.909	1.14 (0.75-1.73)	0.531
Remain sarcopenia	37/150	1.23 (0.86-1.77)	0.265	1.26 (0.87-1.82)	0.222	1.34 (0.93-1.95)	0.118
Deteriorated sarcopenia	140/551	1.52 (1.24-1.86)	<0.001	1.53 (1.25-1.88)	<0.001	1.53 (1.25-1.87)	<0.001
**Heart problems**							
Remain non-sarcopenia	278/2476	Ref	Ref	Ref	Ref	Ref	Ref
Improved sarcopenia	19/139	1.03 (0.64-1.65)	0.920	1.05 (0.65-1.68)	0.856	1.12 (0.70-1.80)	0.68
Remain sarcopenia	30/149	1.32 (0.88-1.99)	0.180	1.35 (0.89-2.04)	0.159	1.41 (0.93-2.14)	0.105
Deteriorated sarcopenia	102/548	1.49 (1.18-1.89)	<0.001	1.50 (1.18-1.90)	<0.001	1.49 (1.17-1.89)	0.001
**Stroke**							
Remain non-sarcopenia	134/2473	Ref	Ref	Ref	Ref	Ref	Ref
Improved sarcopenia	6/139	0.67 (0.29-1.53)	0.337	0.69 (0.30-1.57)	0.371	0.86 (0.38-1.97)	0.713
Remain sarcopenia	10/147	0.94 (0.48-1.86)	0.868	0.97 (0.49-1.94)	0.938	1.09 (0.54-2.17)	0.815
Deteriorated sarcopenia	49/545	1.50 (1.07-2.10)	0.020	1.50 (1.07-2.12)	0.020	1.50 (1.06-2.11)	0.022

Model 1 adjusted for age, and gender.

Model 2 adjusted for age (binary variable: <70 vs. ≥70 years), gender, HB, BMI, marital status, living place, hukou, educational level, smoking status, and drinking status.

Model 3 adjusted for variables in Model 2 and the history of hypertension, diabetes, dyslipidemia, and kidney diseases.

Figure S1 Flowchart of study population selection

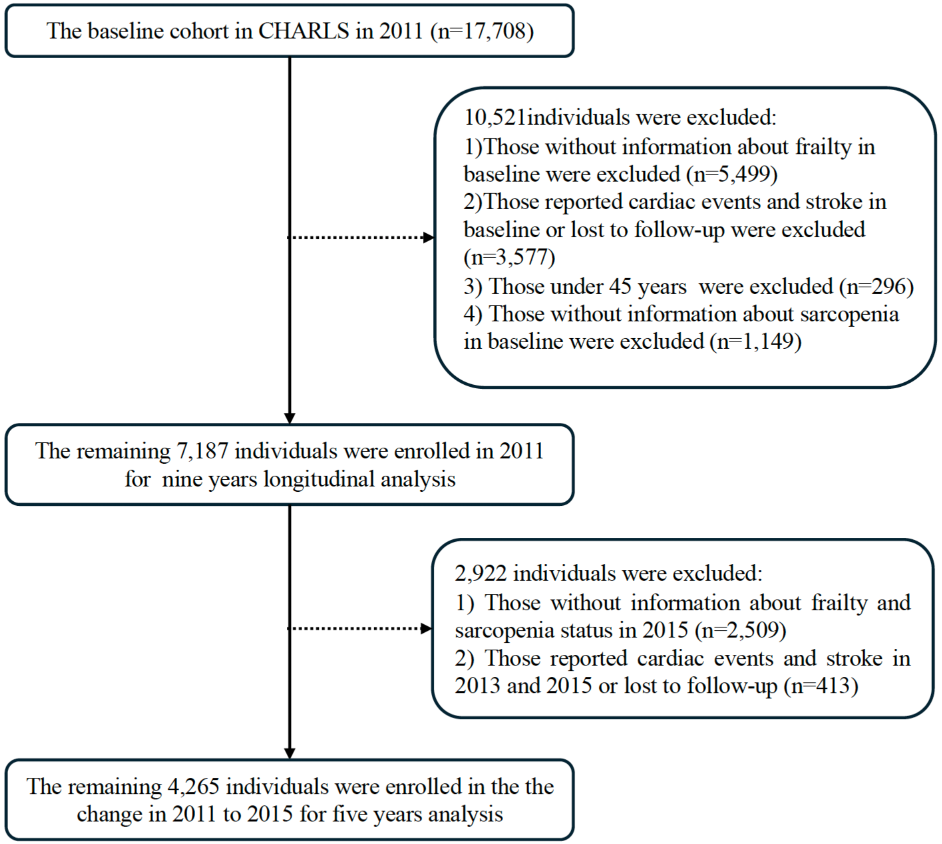

Figure S2 Kaplan-Meier analysis for CVD according to frailty and sarcopenia status

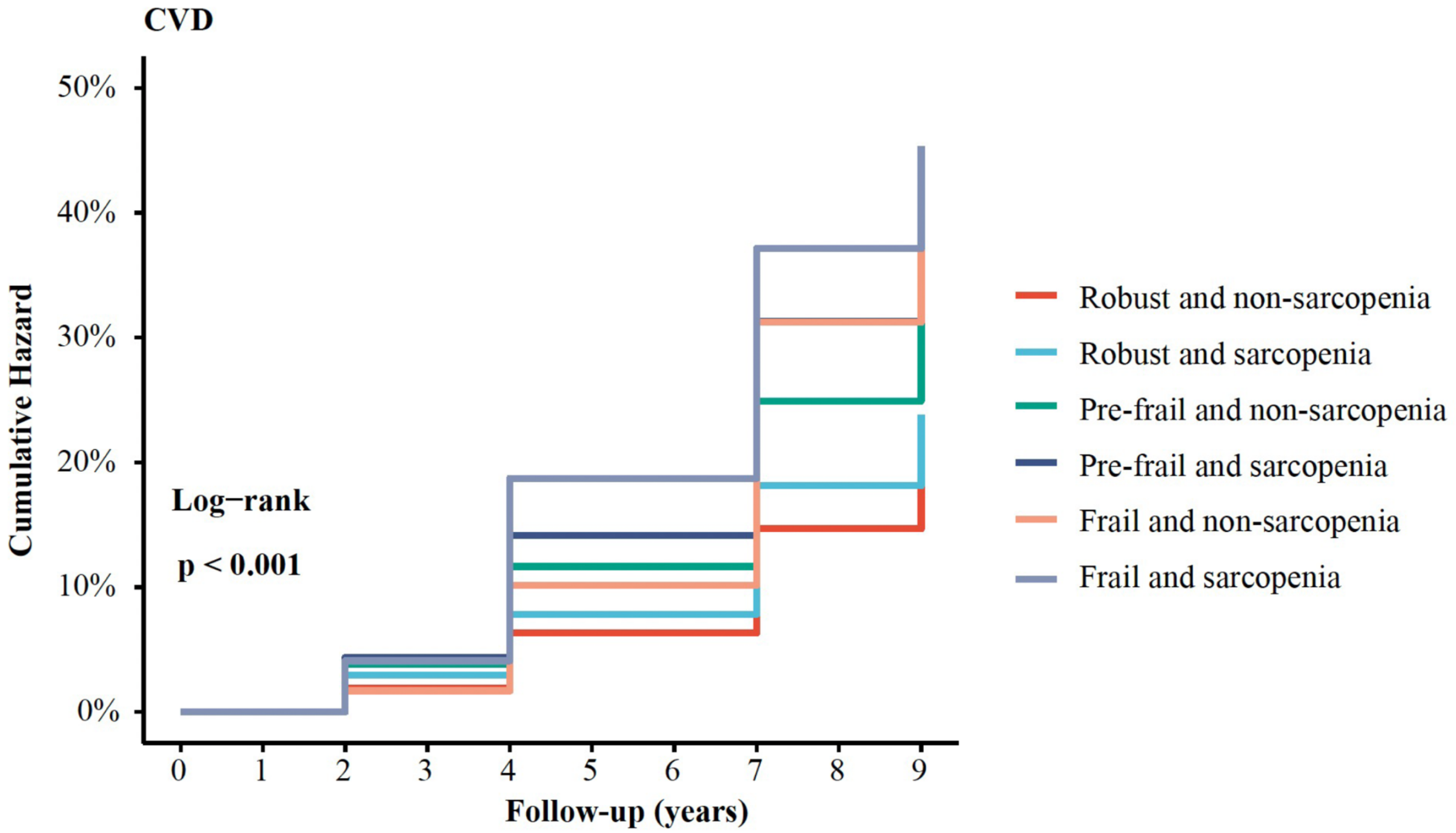

Figure S3 Kaplan-Meier analysis for heart problems (A) or stroke (B) according to frailty and sarcopenia status

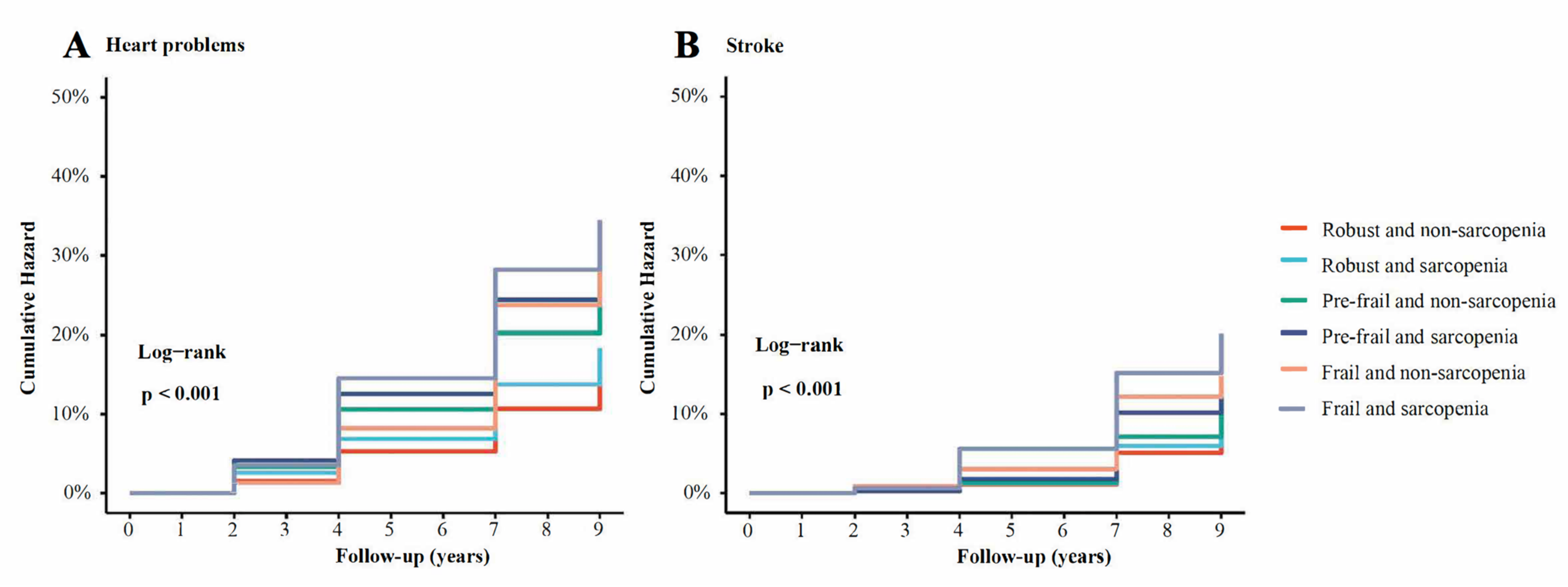

Supplement: Online Supplementary Document [file jogh-15-04299-s001.pdf]
